# Supplementary figures and images for: Reduced MOV10 reveals novel functional cortical connections in an increased fear response
Source: bioRxiv. 2026 May 18:2026.05.18.725995. Preprint. [Version 1] doi: 10.64898/2026.05.18.725995 (PMC13228389; doi:10.64898/2026.05.18.725995)

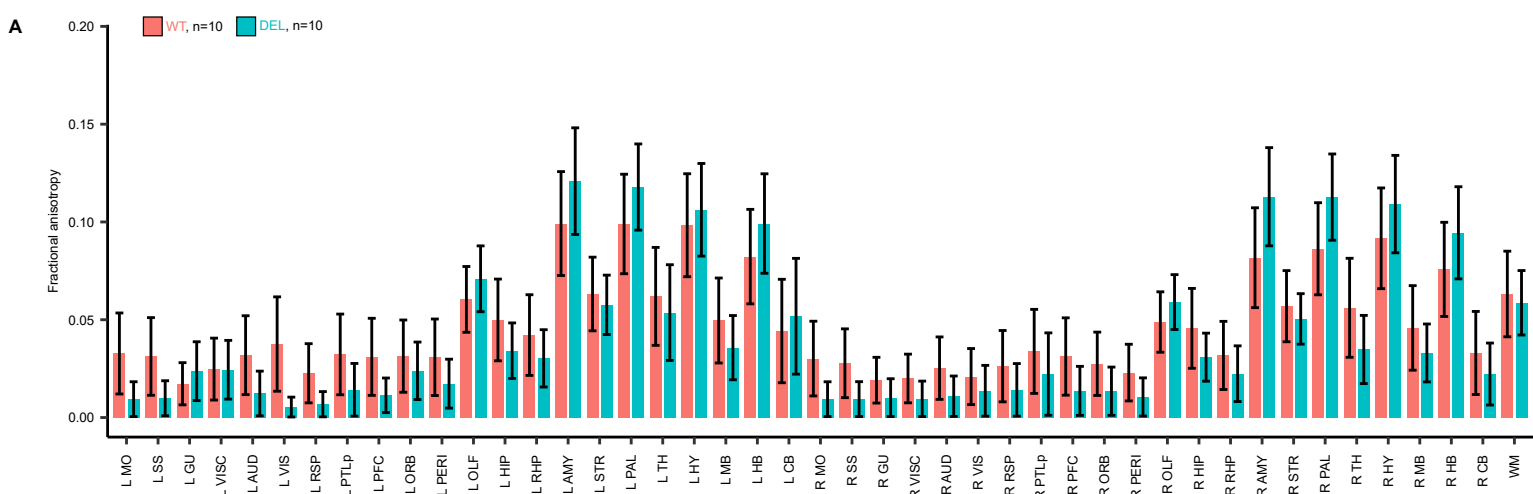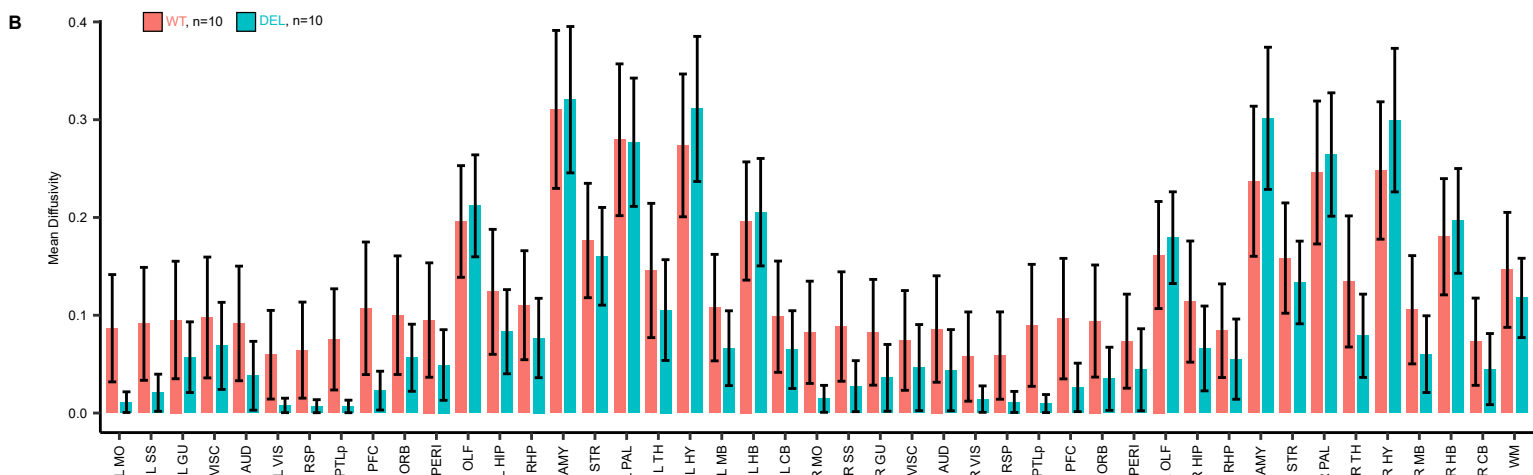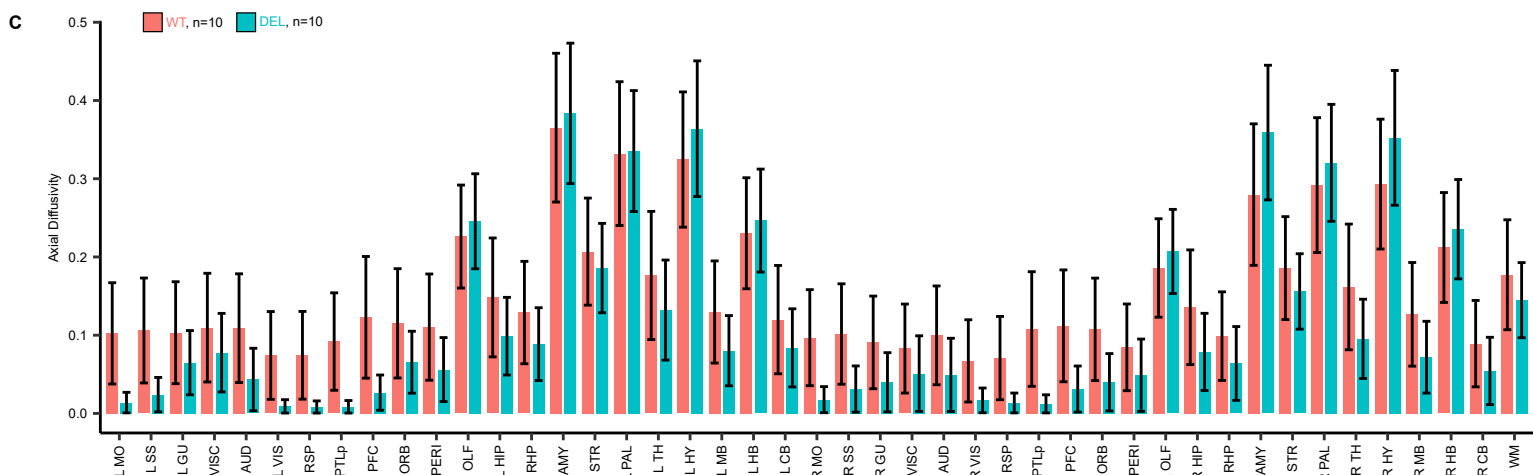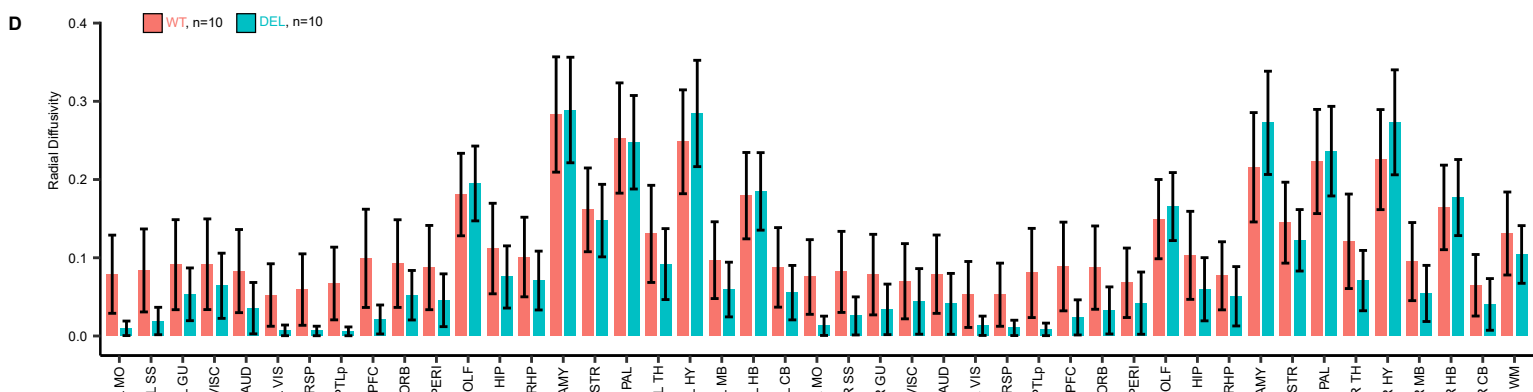

Supplement: Supplement 1 — A) Average fractional anisotropy from n=10 DTI scans of WT and Mov10 Deletion mice across 22 bilateral regions and white matter (WM). B) Average mean diffusivity from n=10 DTI scans of WT and Mov10 Deletion mice across 22 bilateral regions and white matter (WM). C) Average axial diffusivity from n=10 DTI scans of WT and Mov10 Deletion mice across 22 bilateral regions and white matter (WM). D) Average radial diffusivity from n=10 DTI scans of WT and Mov10 Deletion mice across 22 bilateral regions and white matter (WM). Data are shown as mean ± SEM, n is the number of mice of the genotype indicated, of both sexes from N > 3 litters. [file media-1.pdf]

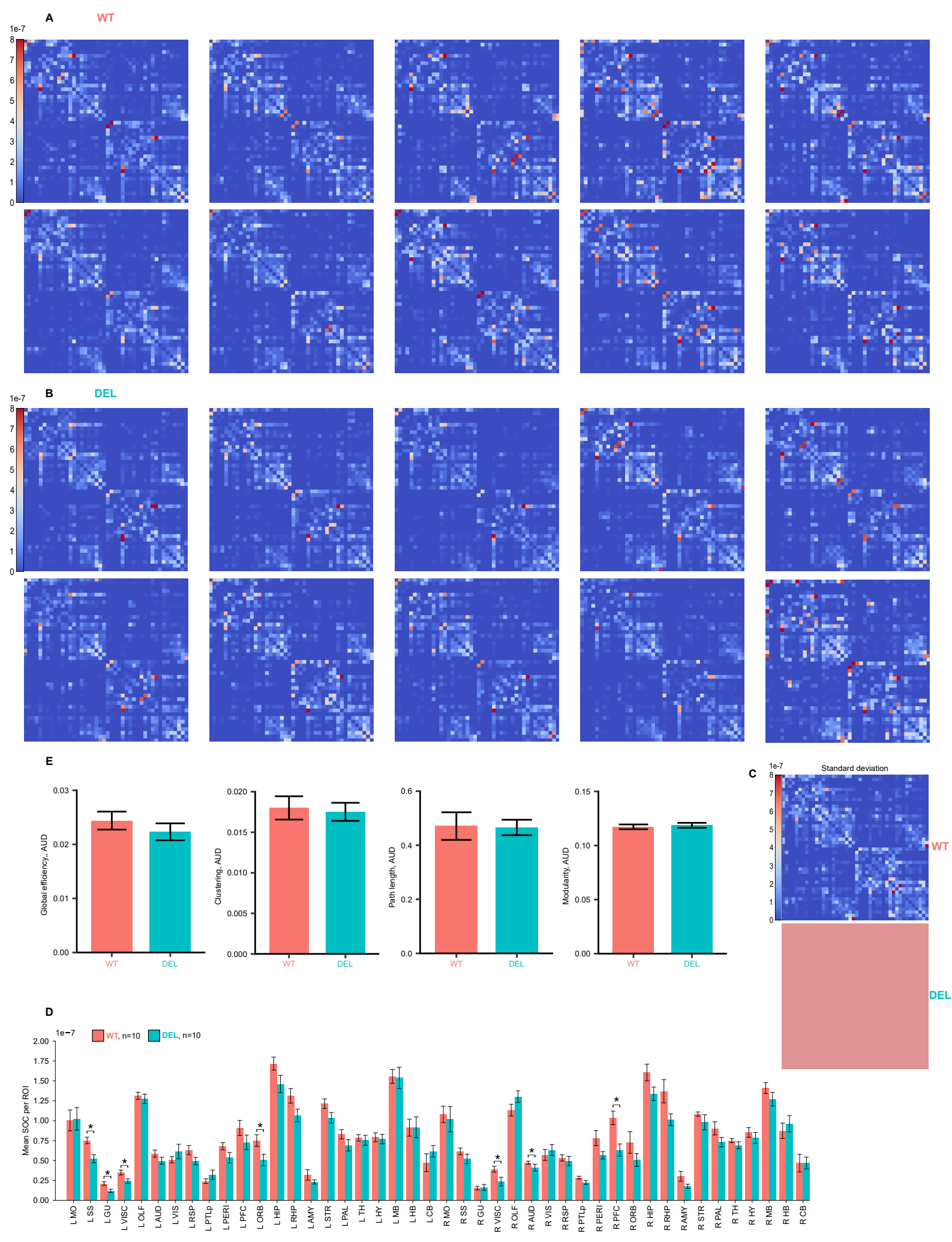

Supplement: Supplement 2 — A) Connectivity matrices with strength of connectivity (SOC) between 22 bilateral brain regions from n=10 DTI scans of WT mice. B) Connectivity matrices with SOC between 22 bilateral brain regions from n=10 DTI scans of Mov10 Deletion mice. C) Standard deviation matrices of SOC between 22 bilateral brain regions across 10 animals for each genotype. D) Mean SOC measures for all regions averaged across the X-axis of the connectivity matrix. E) Area under the curve (AUC) of graph analysis measurements calculated on the connectivity matrices with SOC across several thresholds. Data are shown as mean ± SEM, *p-value<0.05, n is the number of mice of the genotype indicated, of both sexes from N > 3 litters. [file media-2.pdf]

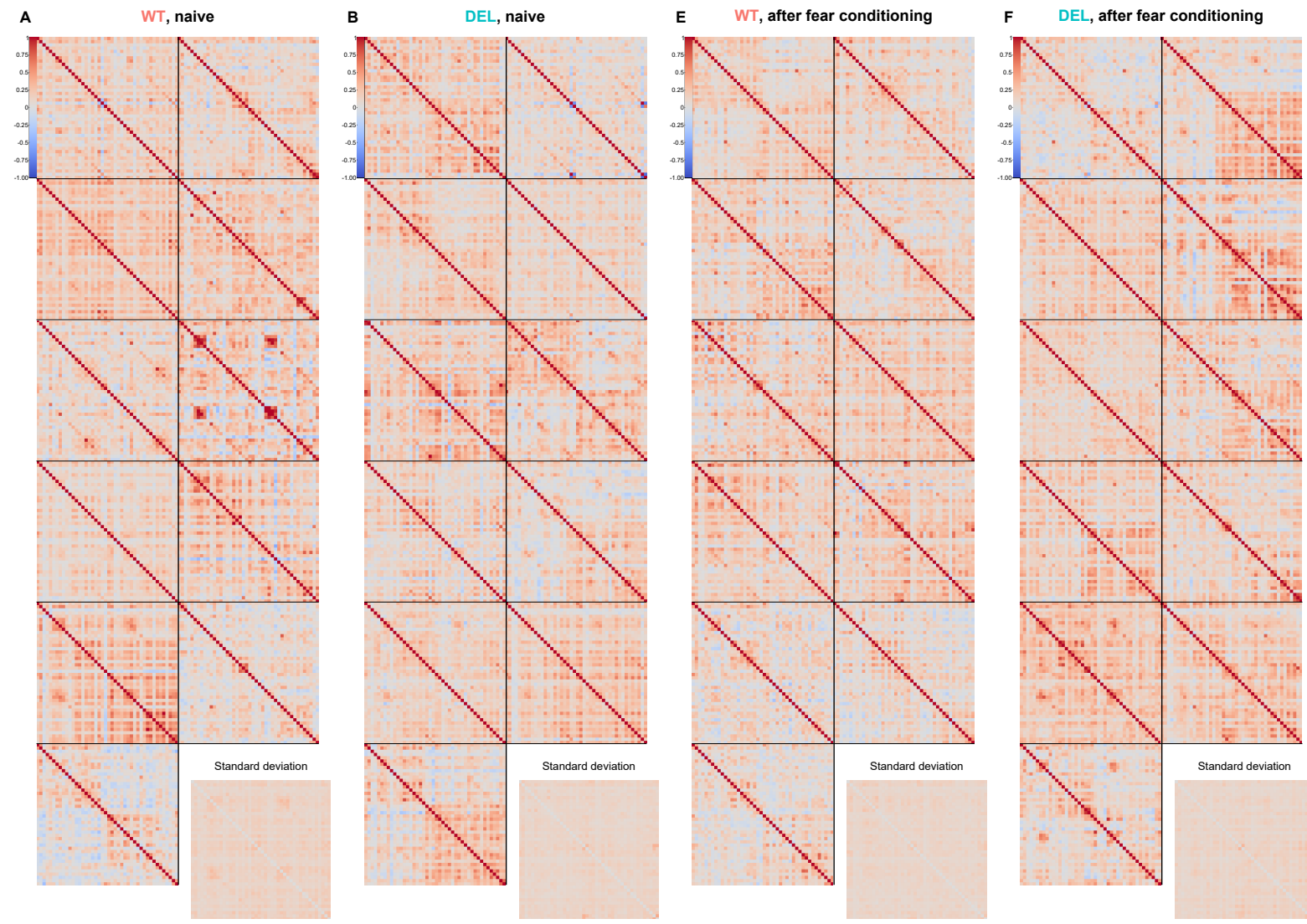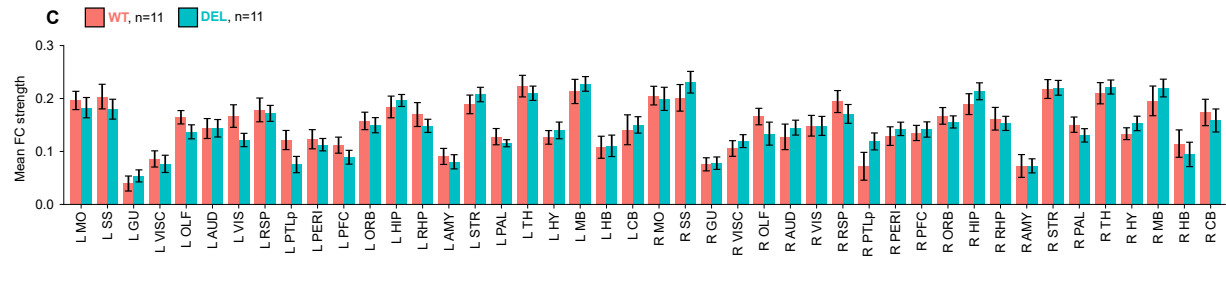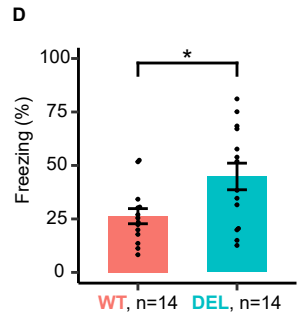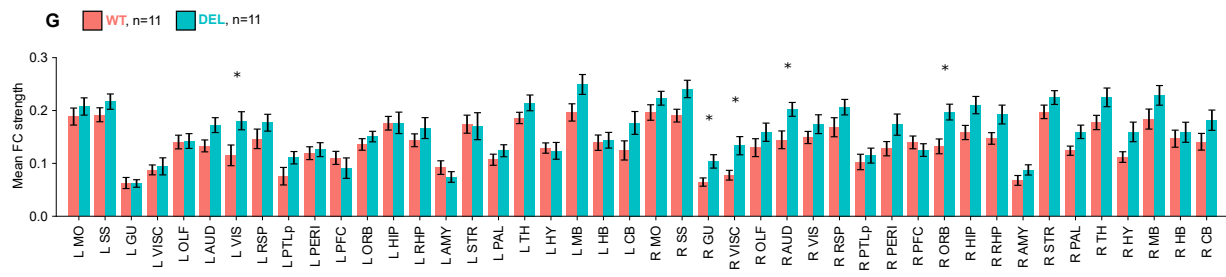

Supplement: Supplement 3 — A) Functional connectivity and standard deviation matrices with Z-transformed Pearson’s correlation coefficients between 22 bilateral brain regions from 14 fMRI scans of naïve WT mice. B) Functional connectivity and standard deviation matrices with Z-transformed Pearson’s correlation coefficients between 22 bilateral brain regions from 14 fMRI scans of naïve Mov10 Deletion mice. C) Mean functional correlation coefficients calculated from rs-fMRI data acquired before fear conditioning averaged across the X-axis of the connectivity matrix. D) Results of the context fear conditioning test on mice that were scanned using MRI. E) Functional connectivity and standard deviation matrices with Z-transformed Pearson’s correlation coefficients between 22 bilateral brain regions from 14 fMRI scans of WT mice after fear conditioning test. F) Mean functional connectivity and standard deviation matrices with Z-transformed Pearson’s correlation coefficients between 22 bilateral brain regions from 14 fMRI scans of Mov10 Deletion mice after fear conditioning. G) Mean functional correlation coefficients calculated from fMRI data acquired before fear conditioning averaged across the X-axis of the connectivity matrix. Data are shown as mean ± SEM, *p-value<0.05, n is the number of mice of the genotype indicated, of both sexes from N > 3 litters. [file media-3.pdf]

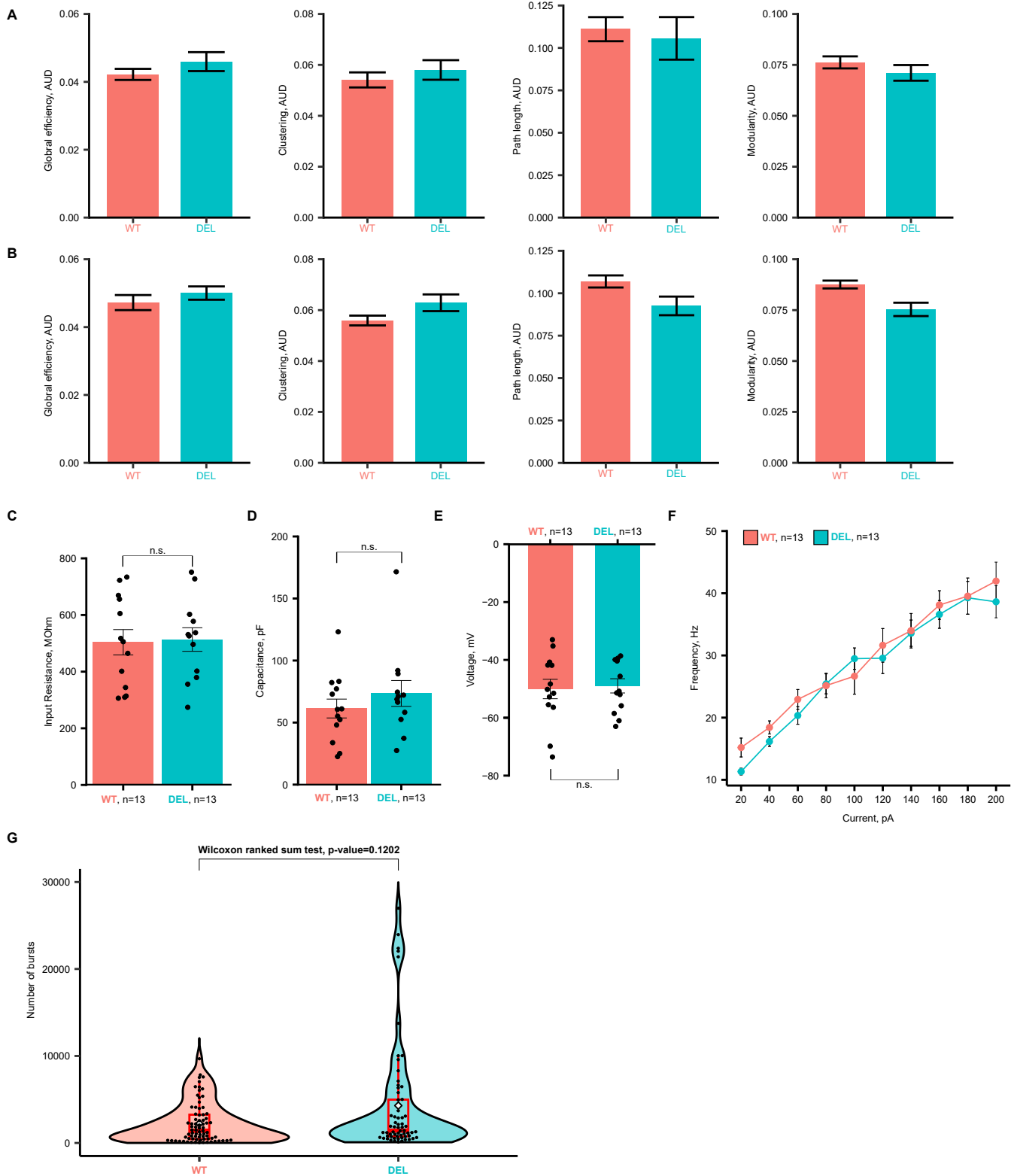

Supplement: Supplement 4 — A) Area under the curve (AUC) of graph analysis measurements calculated on the connectivity matrices with Z-transformed Pearson’s correlation coefficients from fMRI data of naïve mice across several thresholds. B) Area under the curve (AUC) of graph analysis metrics calculated on the connectivity matrices with Z-transformed Pearson’s correlation coefficients from fMRI data of fear conditioned mice across several thresholds. C) Membrane input resistance of WT and Mov10 Deletion (DEL) DIV14 cultured hippocampal neurons. D) Membrane capacitance of WT and Mov10 Deletion DIV14 cultured hippocampal neurons. E) Membrane resting potential of WT and Mov10 Deletion DIV14 cultured hippocampal neurons. F) Instantaneous action potential firing rate for WT and Mov10 Deletion DIV14 cultured hippocampal neurons. Data are shown as mean ± SEM. n is the number of neurons of the genotype indicated, both from N > 3 cultures. G) Number of bursts in baseline recordings of WT and Mov10 Deletion mice. [file media-4.pdf]

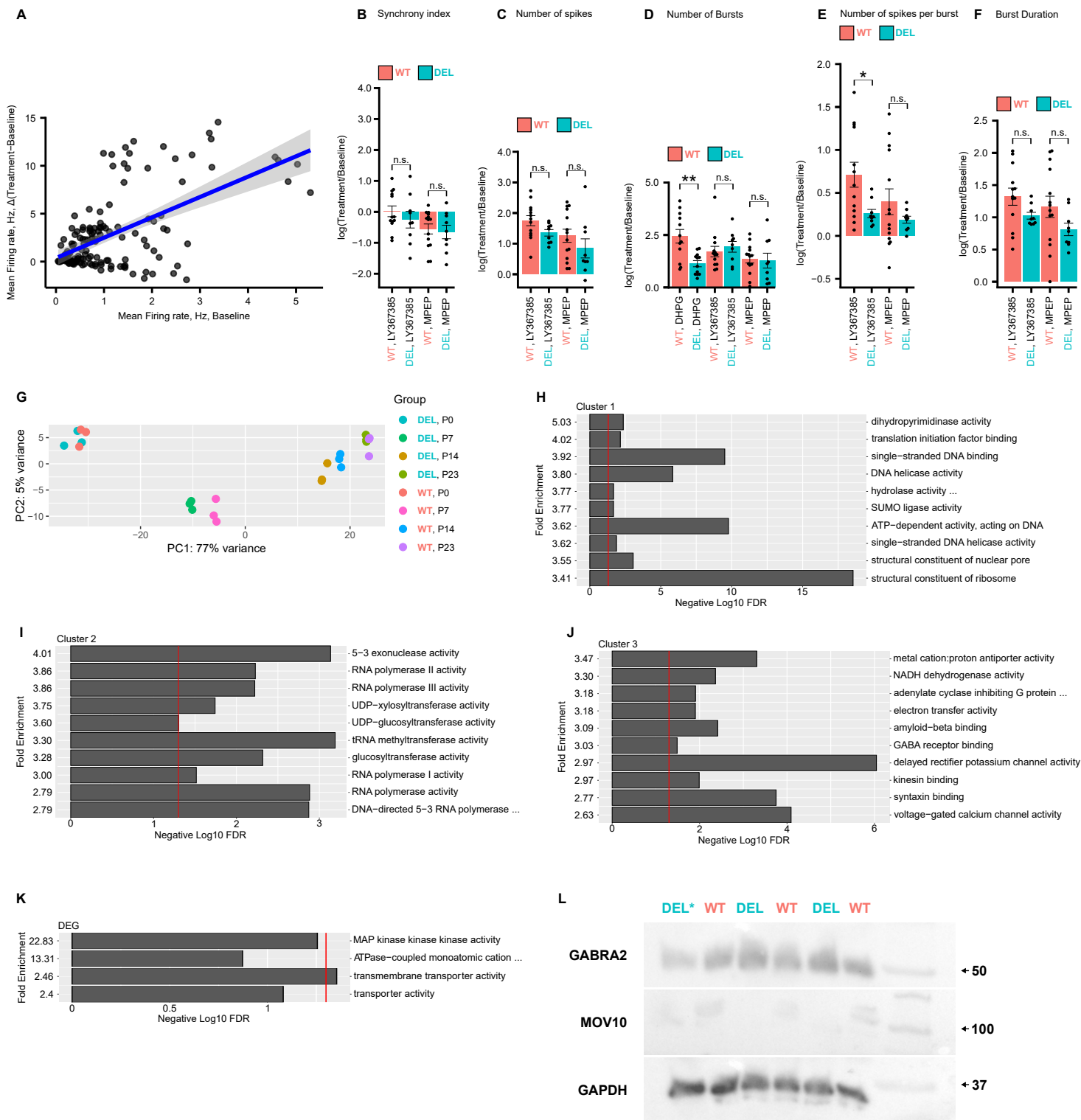

Supplement: Supplement 5 — A) Linear regression of Mean Firing Rate between Δ(Treatment–Baseline) and Baseline. Blue line is the linear fit and shaded area around the line is the standard error. B) Log fold change of synchrony index between Baseline and Treatment for DIV14 WT and Mov10 Deletion (DEL) hippocampal neurons measured across the whole 15 minutes of recording. C) Log fold change of number of spikes between Baseline and Treatment for DIV14 WT and Mov10 Deletion hippocampal neurons measured across the whole 15 minutes of recording. D) Representative raster plots of the last 10 seconds of spiking activity of DIV14 WT and Mov10 Deletion hippocampal neurons before and after bicuculline treatment. Every dash represents a single spike. E) Log fold change of number of bursts between Baseline and Treatment for DIV14 WT and Mov10 Deletion hippocampal neurons measured across the whole 15 minutes of recording. F) Log fold change of number of spikes per burst between Baseline and Treatment for DIV14 WT and Mov10 Deletion hippocampal neurons measured across the whole 15 minutes of recording. G) Log fold change of duration of burst between Baseline and Treatment for DIV14 WT and Mov10 Deletion hippocampal neurons measured across the whole 15 minutes of recording. Data are shown as mean ± SEM from n>4 neuronal cultures from N>3 biological replicates. *p-value<0.05. H-J) Significantly overrepresented gene ontology categories in each of the identified cluster. Red line represents the −log(0.05) = 1.3. K) Gene ontology categories identified in the differentially expressed genes across all timepoints. Red line represents the −log(0.05) = 1.3. L) Immunoblots of GABRA2, MOV10, and GAPDH. Asterisk represents outlier DEL sample that was not included in quantification (Z-score>5). [file media-5.pdf]
